# COVID-19-Induced Guillain-Barré Syndrome

**DOI:** 10.7759/cureus.19809

**Published:** 2021-11-22

**Authors:** Joseph E Brooks, Felicia M Mix, Justin C Buck, Reid A Walters

**Affiliations:** 1 Physical Medicine and Rehabilitation, Genesis Health System, Davenport, USA; 2 Physical Medicine and Rehabilitation, Burrell College of Osteopathic Medicine, Las Cruces, USA; 3 Physical Medicine and Rehabilitation, Lincoln Memorial University-DeBusk College of Osteopathic Medicine, Harrogate, USA; 4 Physical Medicine and Rehabilitation, Olivet Nazarene University, Bourbonnais, USA

**Keywords:** covid induced guillian barre syndrome, electrodiagnostic study, electromyography and electro-stimulation, covid 19, guillain-barre syndrome (gbs)

## Abstract

This case report involves a 47-year-old male who presented to the emergency department (ED) with a positive coronavirus disease 2019 (COVID-19) test and symptoms of Guillain-Barré syndrome (GBS). Electrodiagnostic (EDX) studies reported an acute inflammatory demyelinating polyradiculoneuropathy (AIDP). The patient underwent intravenous immune globulin (IVIG) treatment and four weeks of acute inpatient rehabilitation with some functional improvement but remained unable to ambulate independently at discharge.

## Introduction

Guillain-Barré syndrome (GBS) is an acute autoimmune disorder of the peripheral nerves, first described by Guillain, Barré, and Strohl in 1916 [1]. The onset of GBS is usually preceded by an infection, often of respiratory or gastrointestinal origin, it then clinically presents as rapidly progressing bilateral weakness accompanied by slightly diminished sensation, autonomic dysfunction, and hyporeflexia [2-4]. GBS is often confirmed using electrodiagnostic (EDX) studies and can be further categorized into two main subtypes: acute inflammatory demyelinating polyradiculoneuropathy (AIDP) and acute motor axonal neuropathy (AMAN). While GBS is a relatively rare disease, its prevalence tends to rise during widespread disease outbreaks. Such a phenomenon was observed during the Zika outbreaks of 2013 and 2016 and during a reported epidemic of GBS in the United States in 1976 after patients received the swine flu influenza vaccination [2,3]. This is a case study of a patient who developed GBS following recovery from a coronavirus disease 2019 (COVID-19) infection.

## Case presentation

A 47-year-old male with a past medical history notable for hypertension on metoprolol succinate, morbid obesity, and pre-diabetes presented to the emergency department (ED) with a chief complaint of generalized weakness. The patient tested positive for COVID-19 and exhibited mild unspecified respiratory symptoms. He was subsequently discharged home to recover in isolation per the CDCs COVID-19 response protocols. One week later, the patient returned to the ED for ongoing symptoms and was admitted requiring supplemental oxygen for hypoxia. Upon admission to the hospital, the patient was noted to have difficulty standing and ambulating. Two days into his inpatient stay, the patient developed urinary retention issues requiring intermittent catheterization. Three days later, he developed facial weakness and numbness. The clinical diagnosis of GBS was suspected and subsequent EDX studies reported AIDP. The patient was treated with a five-day course of intravenous immune globulin (IVIG). Three days after the completion of his IVIG treatment, the patient noted improvement with right upper extremity anti-gravity strength. 

The patient was transferred to inpatient rehabilitation with significant proximal lower extremity weakness. The bilateral upper extremities demonstrated slight weakness. The bilateral lower extremities demonstrated a significant loss of strength, 1/5 dorsiflexion, and 3/5 plantar flexion bilaterally. The patient also reported diminished sensation to light touch in bilateral upper extremities in all dermatomes. His blood pressure upon rehabilitation admission was 110/73 mmHg.

Prior to hospitalization, the patient was independent with mobility and all activities of daily living. Upon evaluation in the inpatient rehabilitation, the patient was at a significant functional decline from baseline, requiring dependent assistance with toileting hygiene, showering, upper body dressing, lower body dressing, footwear management, rolling left and right, and all transfers. The patient was unable to ambulate due to his level of impairment. In inpatient rehabilitation, the patient completed three hours total of physical, occupational, and speech therapy per day five days a week with exercises aimed to improve balance, mobility, activities of daily living, fine motor skills, cognition, and breath support.

After completing four weeks of inpatient therapy, the patient was independent with supine to sit, upper and lower body dressings, and rolling right and left in bed. He required minimal assistance to lift the left lower extremity into the bed and moderate assistance with wheelchair-to-bed transfers and bed mobility. He was able to stand with the assistance of his spouse and by using significant reliance on bilateral upper extremity support on a walker for stability and offloading lower extremities. He required a power wheelchair for mobility and was unable to transfer in and out of the car thereby requiring a wheelchair van for transportation. Final muscle strength grading was not documented. His blood pressure remained stable throughout his stay on metoprolol succinate and was 121/65 mmHg on discharge.

## Discussion

GBS is an auto-immune-induced polyneuropathy. The syndrome's etiology is understood to be caused by exposure to foreign peptides similar in structure to peptides found in the myelin and axons in peripheral nerves. B and T cells, unable to differentiate from the pathogenic and human peptides, form an immune response against both, referred to as molecular mimicry [5]. This immune-led assault against the myelin sheath disrupts nerve conduction resulting in a myriad of neurological sequelae. 

In the present case, it is possible that molecular mimicry secondary to COVID-19 infection caused this patient’s sudden onset GBS. This seems especially likely considering the COVID-19 infection was confirmed a week before GBS symptom onset. This hypothesis is further supported by a literature review performed by Abu-Rumeileh et al. which found 53 reports associating COVID-19 with subsequent GBS in a total of 73 patients between January 1, 2020, and July 20, 2020 [6]. Of these 53 reports, eight of the patients were in the United States. Recent research shows that the COVID-19 virus contains hexapeptides (KDKKKK and EIPKEE) that have unique similarities to human heat shock proteins 60 and 90, which are associated with immune-mediated conditions [7]. It is plausible that peptides present in the COVID-19 virus stimulated an auto-immune response in this patient resulting in GBS. 

GBS is diagnosed based on history and physical presentation. However, EDX and cerebrospinal fluid studies can be used as confirmation of the syndrome. The neuropathy induced by GBS often begins with paresthesia and weakness but progresses to paralysis in the distal extremities and ascends proximally [2-4]. In the beginning stages of the syndrome, the patient presented with difficulty ambulating. However, as the syndrome progressed, the patient experienced profound weakness of hip flexors and knee extensors, making unassisted ambulation impossible. 

Patients with GBS often have variable recovery patterns. However, predictions on recovery time can be made with Electromyography (EMG) results. Reductions in compound muscle action potential (CMAP) to less than 20% of normal and prolongation of F-waves on EMG can indicate more advanced GBS and are seen as negative prognostic indicators [8]. Overall, GBS has a favorable prognosis in most cases. Eight out of ten recovering patients will eventually be able to walk unaided, half of whom will do so within a year of diagnosis [9]. The initial rehabilitation goals for patients with GBS involve a reduction in disability burden and preparations for the transition to home life. After initial goals have been met, rehabilitation goals involve reconditioning with the hopes of returning the patient to as close to baseline as possible. Recent literature demonstrates that chronic GBS patients treated with high-intensity therapy with a multidisciplinary ambulatory team, three hours a week, showed considerable improvement in functional outcomes compared to low-intensity, one hour a week, at-home therapy [10]. In the present case, the patient achieved strength and mobility progress throughout his inpatient rehabilitation stay but was unable to ambulate after over four weeks from his initial GBS diagnosis upon discharge. Follow up with therapy and an EDX study is recommended.

## Conclusions

Historically, GBS has been associated with disease outbreaks in the 20th and 21st centuries. The present case is an example of a patient with a positive COVID-19 test developing severe symptoms of GBS one week later. Further research is required to determine a causal relationship between the two pathologies, although we infer our patient’s GBS diagnosis was caused by their COVID-19 infection supported by their EDX study demonstrating an AIDP one week after a positive COVID-19 test. EDX studies are a useful tool in the identification and diagnosis of GBS. Rehabilitation in GBS patients provides increased functional outcomes compared to those who do not participate in therapy. We recommend healthcare providers be aware of the possible relationship between COVID-19 infection and GBS and implement EDX studies for proper diagnosis and expedited treatment including IVIG and comprehensive rehabilitation.

## References

[1] Shoraka S, Ferreira MLB, Mohebbi SR, Ghaemi A (2021). SARS-CoV-2 infection and Guillain-Barré syndrome: a review on potential pathogenic mechanisms. Front Immunol.

[2] Kusunoki S (2016). History of Guillain-Barré syndrome. Clin Exp Neuroimmunol.

[3] Leonhard SE, Mandarakas MR, Gondim FAA (2019). Diagnosis and management of Guillain-Barré syndrome in ten steps. Nat Rev Neurol.

[4] Albers JW, Kelly JJ Jr (1989). Acquired inflammatory demyelinating polyneuropathies: clinical and electrodiagnostic features. Muscle Nerve.

[5] Yuki N, Hartung HP (2012). Guillain-Barré syndrome. N Engl J Med.

[6] Abu-Rumeileh S, Abdelhak A, Foschi M, Tumani H, Otto M (2021). Guillain-Barré syndrome spectrum associated with COVID-19: an up-to-date systematic review of 73 cases. J Neurol.

[7] Lucchese G, Flöel A (2020). SARS-CoV-2 and Guillain-Barré syndrome: molecular mimicry with human heat shock proteins as potential pathogenic mechanism. Cell Stress Chaperones.

[8] Chung T, Prasad K, Lloyd TE (2014). Peripheral neuropathy: clinical and electrophysiological considerations. Neuroimaging Clin N Am.

[9] Rajabally YA, Uncini A (2012). Outcome and its predictors in Guillain-Barre syndrome. J Neurol Neurosurg Psychiatry.

[10] Khan F, Pallant JF, Amatya B, Ng L, Gorelik A, Brand C (2011). Outcomes of high- and low-intensity rehabilitation programme for persons in chronic phase after Guillain-Barré syndrome: a randomized controlled trial. J Rehabil Med.

